# Distal radius reconstruction with vascularized proximal fibular autograft after en-bloc resection of recurrent giant cell tumor

**DOI:** 10.1186/s12891-016-1211-8

**Published:** 2016-08-17

**Authors:** Yun-fa Yang, Jian-wei Wang, Pin Huang, Zhong-he Xu

**Affiliations:** 1 Division of Orthopaedic Trauma and Hand Surgery, Department of Orthopaedic Surgery, Guangzhou First people’s Hospital, Guangzhou Medical University, 1 Panfu Road, Guangzhou, Guangdong 510180 People’s Republic of China; 2 Department of Orthopaedic Surgery, Liwang Hospital, Guangzhou Medical University, Guangzhou, Guangdong 510170 People’s Republic of China

**Keywords:** Distal radius reconstruction, Vascularized proximal fibular graft, Recurrent giant cell tumor, En-bloc resection

## Abstract

**Background:**

Giant cell tumors (GCTs) located in the distal radius are likely to recur, and the treatment of such recurrent tumors is very difficult. Here, we report our clinical experience in distal radius reconstruction with vascularized proximal fibular autografts after en-bloc excision of the entire distal radius in 17 patients with recurrent GCT (RGCT) of the distal radius.

**Methods:**

All 17 patients with RGCT in distal radius underwent plain radiography and/or magnetic resonance imaging (MRI) of the distal radius as the initial evaluation after hospitalization. Then the distal radius were replaced by vascularized proximal fibular autografts after en-bloc RGCT resection. We assessed all patients by using clinical examinations, plain radiography of the wrist and chest, and Mayo wrist scores in the follow-ups.

**Results:**

After an average follow-up of 4.3 years (range: 1.5–10.0 years), no lung metastasis or local recurrence was detected in any of the 17 patients. In total, 14 patients had excellent or good functional wrist scores, 16 were pain free or had occasional pain, and 15 patients returned to work. The mean range of motion of the wrist was 101° (flexion-extension), and the mean grip strength was 77.2 % of the contralateral normal hand.

**Conclusion:**

En-bloc excision of the entire distal radius and distal radius reconstruction with a vascularized proximal fibular autograft can effectively achieve local tumor control and preserve wrist function in patients with RGCT of the distal radius.

## Background

Giant cell tumor (GCT), a type of primary benign bone tumor, is relatively common and usually involves the metaphyseoepiphyseal region of long bones in the extremities [1]. GCT typically occurs in persons under the age of 40 years, and results in mild symptoms, which may continue for months before the patient visits an orthopedist; some GCT patients see a doctor for the first time because of acute pain caused by pathological fractures [2]. GCT is a potentially aggressive bone tumor with the ability to metastasize; as a result, GCT with pulmonary metastasis is occasionally detected at the very first clinical examination.

GCT commonly involves the distal femur and proximal tibia. GCT in the distal radius is also common and difficult to treat. Intralesional curettage with bone-graft fillings is an acceptable primary treatment for GCT of the radius; however, GCT has a relatively high rate of local recurrence after curettage, especially, GCT in the distal radius [3, 4]. Furthermore, GCT in the distal metaphyseoepiphyseal region of the radius is commonly associated with extracompartmental extension, cortical invasion, and pathologic fracture. Therefore, curettage is not a rational method for the management of primary GCT in the distal radius, let alone recurrent GCT (RGCT) in this site. Fortunately, en-bloc resection with some type of reconstruction surgery, ranging from arthrodesis to structural bone allograft/autograft replacement or wrist arthroplasty, which may reduce the rate of recurrence, can be effective in treating RGCT in the distal radius [3, 5, 6].

Wrist arthroplasty is not the first choice of reconstruction surgery because most of these patients are young and active, and want to retain a functional wrist [7, 8]. En-bloc resection of the tumor followed by structural fibular allograft or non-vascularized fibular autograft for the reconstruction of the distal part of the radius is much better than curettage [9]. Unfortunately, it also has many complications such as nonunion, wrist instability, recurrence, and the complication rates associated with such reconstruction of distal radius are universally high (even more than 50 %, rang from 0 to 66.6 %) [9]. Therefore, we think that the results of the reconstruction with a fibular allograft or non-vascularized fibular autograft are not suitable in the distal radius RGCT cases, because of nonunion caused by insufficient blood supply, wrist instability or potential wrist collapse secondary to bone absorption.

To overcome these drawbacks, we devised our clinical strategy of en-bloc resection and reconstruction of the entire distal radius with a vascularized proximal fibular autograft for the treatment of RGCT in the distal radius. Herein, we present the outcomes of this surgical strategy.

## Methods

This study was approved by ethics committee of our hospital, and all patients were well informed of this study after hospitalization. A total of 17 patients with histologically proven RGCT (Campanacci grade II or III) in the distal radius were treated with en-bloc resection and reconstruction with a vascularized proximal fibula between 2003 and 2012 in our department.

### Patients

This study involved 12 men and five women with a mean age of 23.2 years (range: 19–48 years). All 17 patients underwent plain radiography and/or magnetic resonance imaging (MRI) of the distal radius as the initial evaluation after hospitalization. Chest X-rays and/or chest computed tomography (CT) scans were required in the first evaluation. All the patients had been treated using no less than one surgery with intralesional curettage, and the bone cavities had been filled with a bone allograft/autograft. None of the patients had lung metastases at the time of enrollment in our study. The RGCT was detected an average of 14 months (range: 3 months to 3 years) after the curettage surgery. The lesion was in the right radius in 11 patients and in the left radius in six patients. All 17 patients attended follow-up for at least 1.5 years.

### Surgical technique

#### En-bloc resection of the entire distal radius

The precise margins of the tumor were ascertained using plain radiography and/or MRI. A safe surgical margin was defined as a distance of no less than 2.5 cm from the bony involvement, and the appropriate length of the vascularized proximal fibular graft for distal radius reconstruction was determined accordingly [8]. The site of the RGCT in the distal radius was approached directly via a palmar radial incision. The radial artery, radial veins, and cephalic vein were identified and protected during the surgical exposure of the distal radius. To maintain wrist stability, we retained as much of the distal radioulnar joint (DRUJ) capsule and the radiocarpal ligaments as possible for DRUJ reconstruction. After the bony resection, we carefully measured the amount of bone resected (bone defect size: 6.4 ± 1.2 cm) and prepared the recipient bone ends.

#### Harvesting of vascularized proximal fibular grafts

We harvested the ipsilateral proximal fibula to better match the shape of the distal radius. We made an approximately 15 cm curved and longitudinal skin incision, which was almost parallel to the fibula and centered on the fibular head, beginning 7 cm above the fibular head and about 1 cm behind the fibula, extending towards the distal fibula, and ending in the proximal 1/3rd of the leg. First, we carefully identified and protected the inferior lateral genicular vessels, and then located the peroneal vessels and their branches by identifying the intermuscular septa between the gastrocnemius, soleus, tibialis posterior, and flexor hallucis longus. Then, we retained a muscle sleeve around the periosteum to avoid peroneal vascular injury during the surgery. The biceps femoris tendon, fibular collateral ligament, capsule around the fibular head, common peroneal nerve, superficial peroneal nerve, deep peroneal nerve, and popliteal vessels were carefully localized intraoperatively [8]. Because of the perfusion characteristics of the proximal fibula in the adult, we confirmed that the bleeding of the soft tissue attached around the fibular head was satisfactory, and then harvested the proximal fibula along with the inferior lateral genicular vessels and/or peroneal vessels. The length of the fibular graft depended on the bone defect secondary to the distal radius resection. Finally, we reconstructed the remnant soft-tissue, including the biceps femoris tendon, fibular collateral ligament, and proximal tibiofibular joint capsule to maintain the lateral stability of the knee.

#### Reconstruction of the distal radius

We transplanted the proximal fibula to replace the entire distal radius after en-bloc resection of the RGCT. First, we inserted the proximal fibular graft in the place of the distal radius and confirmed the appropriateness of the replacement by using intraoperative C-arm X-ray examination. We selected a plate and/or screws for skeletal fixation. We usually separated the biceps femoris tendon into three bundles for reconstruction, in order to ensure the dorsal and palmar stability of the DRUJ and the lateral stability of the wrist after fibular transplantation. Then, we sutured the capsule around the fibular head and the biceps femoris tendon with the remnant of the DRUJ capsule and the radiocarpal ligaments (for DRUJ stability) and radial collateral ligament (for lateral stability of the wrist). The inferior lateral genicular artery and vein or peroneal artery and vein were connected via an end-to-end and/or end-to-side anastomosis with the radial artery and veins or one of the accompanying radial veins and the cephalic vein. After confirming that the soft tissue around the fibular head had an active blood supply, we closed the incision. Finally, we fixed the elbow and wrist in a functional position (90°of elbow flexion and 20° of wrist extension) with a long-arm plaster splint [8].

### Postoperative evaluation

We monitored bone healing by comparing preoperative and follow-up radiographs. We removed the splint 6 weeks after the surgery, at which time, the patient was permitted to perform gentle range-of-motion exercises. During follow-up clinical examinations, we evaluated wrist function by using the four-item Mayo wrist scoring system, which includes the items pain intensity, functional status, range of motion, and grip strength [10]. We removed the internal fixation 18 months after the surgery. We also evaluated the complications in the donor knee.

## Results

The average follow-up duration was 4.3 years (range: 1.5–10.0 years). All 17 patients had achieved bony union between 3 and 5 months after the surgery. None of the patients had a GCT recurrence by the time of the last follow-up. Clinical assessments did not reveal lung metastases in any patient. No patient had discomfort at the proximal fibula donor site, and no patient complained of lateral instability of the knee. Neither postoperative infections nor neurovascular complications occurred in the patients.

Nine patients were pain free (52.9 %), five had mild and occasional pain (29.5 %), three had moderate but tolerable pain (17.6 %), and no patient had intolerable pain. Nine patients returned to their previous work without any limitations (52.9 %), 6 returned to work but with a little restriction (35.3 %), two patients were able to work but were unemployed (11.8 %), and no patient was disabled due to the RGCT after the reconstructive surgery.

All patients showed some limitation in the range of motion of the wrist. The flexion-extension range was between 90° and 145° in eight patients (47.1 %), between 60° and 90° in six patients (35.3 %), and between 30° and 60° in three patients (17.6 %). No patient had a flexion-extension range of less than 30°. The mean range of motion at the wrist (flexion/extension) was 101° (flexion, 49°; extension, 52°); the normal wrist extension/flexion range of motion is 70°–75°.

Compared with the normal hand, the affected hand had a grip strength of 75–100 % in 11 patients, 50–75 % in five patients, and 25–50 % in one patient. No patient had a grip strength of 100 % or less than 25 % of the normal hand. The mean grip strength of the affected hand was 77.2 % of the normal hand.

**Fig. 1:**
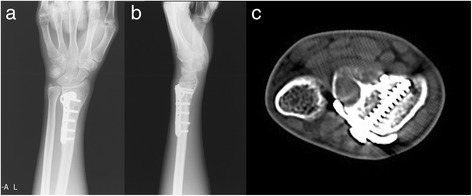
Preoperative anteroposterior and lateral X-ray and CT scans of a 20-year-old man with RGCT of the left distal radius (Campanacci grade III, recurrence at 13 months after curettage) (**a**-**c**)

**Fig. 2:**
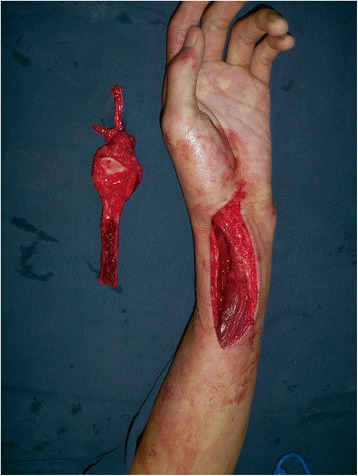
Harvesting of the vascularized proximal fibula and en-bloc resection of the entire distal radius

**Fig. 3:**
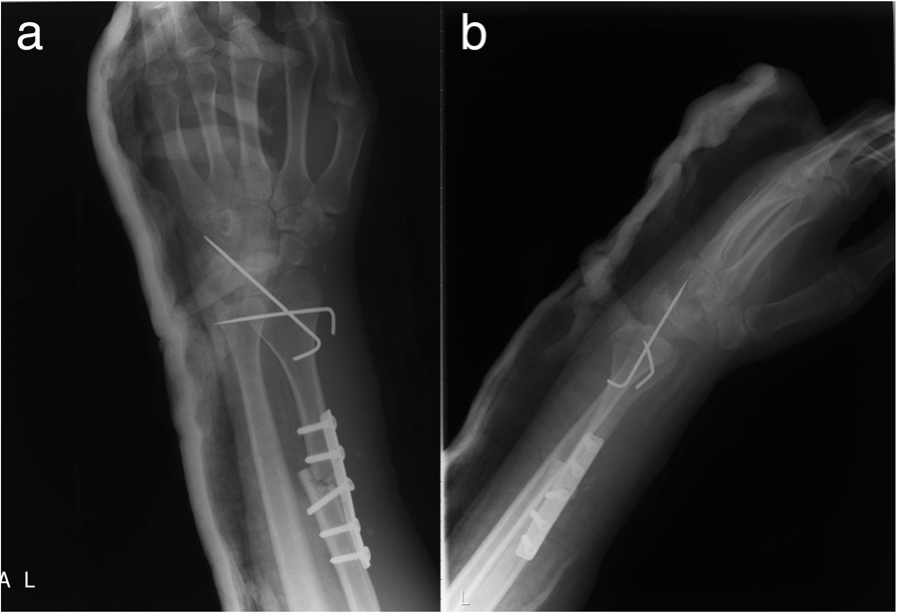
Anteroposterior and lateral X-ray images taken 1 week after en-bloc resection of RGCT and wrist reconstruction with a vascularized proximal fibular autograft (**a**, **b**)

**Fig. 4:**
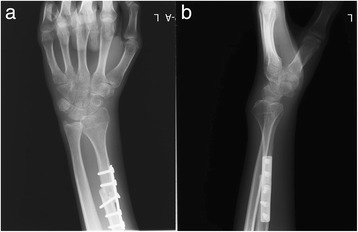
Anteroposterior and lateral X-ray images of the wrist taken 3 months after the reconstruction (**a**, **b**)

**Fig. 5:**
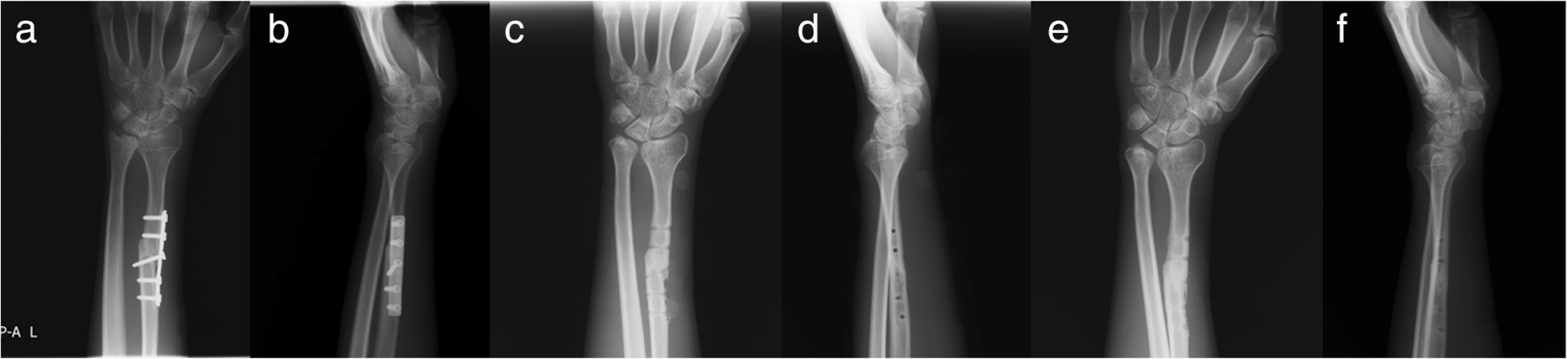
Anteroposterior and lateral X-ray images taken 18 months after wrist reconstruction, before and after the removal of internal fixation (**a**-**d**), and images taken 30 months after wrist reconstruction (**e**, **f**)

According to the Mayo wrist scores, nine patients (52.9 %) had excellent outcomes, six (35.3 %) had good outcomes, 1 (5.9 %) had a moderate outcome, and one patient (5.9 %) had a poor outcome. The mean wrist score was 77.3 (Figs. 1, 2, 3, 4 and 5).

## Discussion

GCT is a potentially aggressive benign tumor, and is associated with a high rate of local recurrence and a risk of pulmonary metastasis [11]. Local recurrence of GCT is usually attributable to tumor cells remaining behind or being implanted in the surgical site [12]. Of these two possibilities, recurrence due to tumor cell contamination of the surgical site by the instruments used during the surgery is less likely [13].

Local control of RGCT is difficult, especially, when the tumor is located at the end of the radius [9, 14, 15]. Therefore, the principal RGCT treatment should consist of the complete removal of the lesion, reduction of the risk of recurrence, and preservation of limb function [15]. Some authors report that over 80 % patients have local recurrence after the curettage or cementing of GCTs located in the distal radius [3]. For the local control of GCTs of the distal radius and preservation of wrist function, en-bloc resection and wrist reconstruction seems to be an effective method. Many reconstruction procedures, including arthroplasty, osteoarticular allograft, allograft arthrodesis, and vascularized or non-vascularized fibular autograft with or without arthrodesis, have been proposed after wide resection of the distal radius [15–23]. However, because of the incompatibility between prostheses and the host bone, prostheses are not suitable for long-term survival. To ensure long-term survival of the prosthesis, bone union is the most reliable method. Thus, bone grafts are still the first choice for repairing distal radius defects secondary to RGCT resection. All bone allografts have disadvantages, including lack of blood supply and osteogenic cells, potential immunologic reactions, difficulty in DRUJ reconstruction, and possibility of wrist collapse secondary to bone allograft absorption. Therefore, bone allografts are not the best choice for wrist reconstruction.

Non-vascular fibular autografts can maintain the anatomy of the wrist and preserve wrist function, and are free from viral transmissions. However, these grafts are associated with many complications (such as nonunion) and do not result in very satisfactory outcomes [9, 17, 20, 22]. We have the experience of non-vascular fibular autografts collapse in reconstruction of distal radium, since then, we never use non-vascular fibular autografts for distal radium reconstruction. Vascularized fibular autografts have the same donor complications as non-vascularized fibular autografts, but have an intrinsic blood supply and live bone cells. These vascularized autografts also have the ability to undergo osteogenesis and yield well-perfused bones. The biological advantage of vascularized fibular autografts is that the healing process between the fibula and the host is identical to that observed in normal fracture healing, without any evidence of creeping substitution and immunologic reactions. Therefore, the union time of these grafts is much shorter than that of non-vascularized bone grafts or allografts, and may be as short as 3–5 months. Hence, the vascularized proximal fibula may be the best replacement for the distal radius after en-bloc excision of RGCT of the distal radius.

In this study, there were no cases of lung metastasis or bony recurrence, as determined using clinical assessments, by the time of the last follow-up. The mean Mayo wrist score was 77.3. Of the 17 patients, 82.4 % had excellent or good results, 82.4 % had no pain or only occasional pain, and 88.2 % returned to work. Although all patients had some limitation in the range of motion of the wrist, the mean range of motion was 75.5 % of the normal wrist. The mean grip strength was 77.2 % of the normal contralateral hand.

Although our findings indicate that reconstruction of the entire distal radius with a vascularized proximal fibula after RGCT resection is feasible, effective, and reliable, there are some weaknesses and limitations of our study. First, the operation procedure is very complicated because it requires microsurgical techniques. Second, because of the small sample size, the study findings need to be confirmed using further clinical data and long-term follow-up. Third, wrist function may be severely limited after the reconstruction because the proximal fibula cannot exactly match the distal radius.

## Conclusion

En-bloc resection of RGCT of the distal radius and reconstruction with a vascularized proximal fibular autograft are effective for local tumor control and wrist-function preservation.

## Abbreviations

DRUJ: Distal radioulnar joint
GCTs: Giant cell tumors
RGCT: Recurrent GCT
